# The systems biology simulation core algorithm

**DOI:** 10.1186/1752-0509-7-55

**Published:** 2013-07-05

**Authors:** Roland Keller, Alexander Dörr, Akito Tabira, Akira Funahashi, Michael J Ziller, Richard Adams, Nicolas Rodriguez, Nicolas Le Novère, Noriko Hiroi, Hannes Planatscher, Andreas Zell, Andreas Dräger

**Affiliations:** 1 Center for Bioinformatics Tuebingen (ZBIT), University of Tuebingen, Tübingen, Germany; 2 Graduate School of Science and Technology, Keio University, Yokohama, Japan; 3 Department of Stem Cell and Regenerative Biology, Harvard University, Cambridge, MA, USA; 4 SynthSys Edinburgh, CH Waddington Building, University of Edinburgh, Edinburgh EH9 3JD, UK; 5 European Bioinformatics Institute, Wellcome Trust Genome Campus, Hinxton, Cambridge, UK; 6 Babraham Institute, Babraham, Cambridge, UK; 7 Present address: Natural and Medical Sciences Institute at the University of Tuebingen Reutlingen, Germany; 8 Present address: University of California, San Diego, 417 Powell-Focht Bioengineering Hall 9500, Gilman Drive, La Jolla, CA 92093-0412, USA

**Keywords:** Systems biology, Biological networks, Mathematical modeling, Simulation, Algorithms, Ordinary differential equation systems, Numerical integration, Software engineering

## Abstract

**Background:**

With the increasing availability of high dimensional time course data for metabolites, genes, and fluxes, the mathematical description of dynamical systems has become an essential aspect of research in systems biology. Models are often encoded in formats such as SBML, whose structure is very complex and difficult to evaluate due to many special cases.

**Results:**

This article describes an efficient algorithm to solve SBML models that are interpreted in terms of ordinary differential equations. We begin our consideration with a formal representation of the mathematical form of the models and explain all parts of the algorithm in detail, including several preprocessing steps. We provide a flexible reference implementation as part of the Systems Biology Simulation Core Library, a community-driven project providing a large collection of numerical solvers and a sophisticated interface hierarchy for the definition of custom differential equation systems. To demonstrate the capabilities of the new algorithm, it has been tested with the entire SBML Test Suite and all models of BioModels Database.

**Conclusions:**

The formal description of the mathematics behind the SBML format facilitates the implementation of the algorithm within specifically tailored programs. The reference implementation can be used as a simulation backend for Java™-based programs. Source code, binaries, and documentation can be freely obtained under the terms of the LGPL version 3 from http://simulation-core.sourceforge.net. Feature requests, bug reports, contributions, or any further discussion can be directed to the mailing list simulation-core-development@lists.sourceforge.net.

## Background

As part of the movement towards quantitative biology, the modeling, simulation, and computer analysis of biological networks have become integral parts of modern biological research [1]. Ambitious national and international research projects such as the Virtual Liver Network [2] strive to derive even organ-wide models of biological systems that include all kinds of processes taking place at several levels of detail. Large-scale efforts like this require intensive collaboration between various research groups, including experimenters, modelers, and bioinformaticians. The exchange, storage, interoperability, and the possibility to combine models have been recognized as key aspects of this endeavor [3-6].

XML-based standard description formats such as the Systems Biology Markup Language (SBML) [7,8] and CellML [9,10] enable encoding of quantitative biological network models. To facilitate sharing and re-use of the models, online databases such as BioModels Database [11] and the CellML model repository [12] provide large collections of published models. Software libraries for reading and manipulating the content of these formats are also available [13-15] as well as end-user programs supporting these model description languages.

The models encoded in these formats can be interpreted in terms of several modeling frameworks, including, but not limited to, differential equation systems, with additional structures such as discrete events and algebraic equations. The diversity of modeling approaches and experimental data often requires customized software solutions for very specific tasks. For efficient analysis, simulation, and calibration (e.g., the estimation of parameter values) of biological network models a multiple-purpose and efficient numerical solver library is prerequisite. Although the language specifications of SBML [16-22] and CellML [23] describe the semantics of models in these formats and their interpretation, the algorithmic implementation is still not straightforward.

The SBML community offers standardized and manually derived benchmark tests [24] in order to evaluate the quality of simulation results, because it has been recognized that in many cases different solver implementations lead to divergent results [25]. The availability of this test suite and the currently much larger variety of supporting software for SBML^a^ in comparison to CellML are the reasons that in this work we focus on the simulation of models encoded in the SBML format.

We address the question of how to precisely interpret these models in terms of ordinary differential equation systems. Furthermore, we show how to adapt existing numerical integration routines in order to simulate these models. To this end, we derive a new algorithm for the accurate interpretation and simulation of *all* currently existing levels and versions of SBML. To demonstrate the usefulness of the algorithm, we introduce an exhaustive reference implementation in Java™. The algorithm described in this paper is, however, not limited to any particular programming language.

It is also important to note that the interpretation of these models must be strictly separated from the numerical method that solves the implied differential equation system. In this way, a similar approach would also be possible for other systems biology community formats. In particular, the architecture of the reference implementation described herein has been *ab ovo* designed with the aim to be complemented by a CellML module.

As the result, we present the Systems Biology Simulation Core Library, a platform-independent, well-tested generic open-source library. The library is completely decoupled from any graphical user interface and can therefore easily be integrated into third-party programs. It comprises several ordinary differential equation (ODE) solvers and an interpreter for SBML models. It is the first simulation library based on JSBML [15].

Furthermore, the Systems Biology Simulation Core Library contains classes to both export simulation configurations to the Simulation Experiment Description Markup Language (SED-ML) [26], and facilitate the re-use and reproduction of these experiments by executing SED-ML files.

## Results and discussion

In order to derive an algorithm for the interpretation of SBML models in a differential equation framework, it is first necessary to take a closer look at the mathematical equations implied by this data format. Based on this general description, we will then discuss all necessary steps to deduce an algorithm that takes all special cases for the various levels and versions of SBML into account.

### A formal representation of models in systems biology

The mathematical structure of a reaction network comprises a stoichiometric matrix **N**, whose rows correspond to the reacting species $\vec{S}$ within the system, whereas its columns represent the reactions, i.e., bio-transformations, in which these species participate. The velocities $\vec{\nu }$ of the reaction channels $\vec{R}$ determine the rate of change of the species’ amounts:

(1) $$\frac{d}{dt}\vec{S}=N\vec{\nu }(\vec{S},t,N,W,\vec{p})\;.$$

The parameter vector $\vec{P}$ contains rate constants and other quantities that influence the reactions’ velocities. According to Liebermeister *et al.*[27,28] the modulation matrix **W** is defined as a matrix of size $\left|\vec{R}|\times |\vec{S}\right|$ containing a numerical representation of the type of the regulatory influences of the species on the reactions, e.g., competitive inhibition or physical stimulation. Integrating the homogeneous ordinary differential equation system (1) yields the predicted amounts of the species at each time point of interest within the interval [*t*_0_,*t*_*T*_]:

(2) $$\vec{S}=\overset{t_{T}}{\underset{t_{0}}{\int }}N\vec{\nu }(\vec{S},t,N,W,\vec{p})dt,$$

where $t_{0}\in \mathbb{R}$ and *t*_0_<*t*_*T*_. Depending on the units of the species, the same notation can also express the change of the species’ concentrations. In this simple case, solving equation (2) can be done in a straightforward way using many (numerical) differential equation solvers. The nonlinear form of the kinetic equations in the vector function $\vec{\nu }$ constitutes the major difficulty for this endeavor and is often the reason why an analytical solution of these systems is not possible or very hard to achieve. Generally, differential equation systems describing biological networks are, however, inhomogeneous systems with a higher complexity. Solving systems encoded in SBML can be seen as computing the solution of the following equation:

(3) $$\;\vec{Q}=\overset{t_{T}}{\underset{t_{0}}{\int }}N\vec{\nu }(\vec{Q},t,N,W,\vec{p})+\vec{g}(\vec{Q},t)\text{dt}+{\vec{f}}_{E}(\vec{Q},t)+\vec{r}(\vec{Q},t),$$

with *t*_0_≡0 and $t_{T}\in {\mathbb{R}}_{+}$. The vector $\vec{Q}$ of quantities contains the sizes of the compartments $\vec{C}$, amounts (or concentrations) of reacting species $\vec{S}$, and the values of all global model parameters $\vec{P}$. It should be noted that these models may contain local parameters $\vec{P}$ that influence the reactions’ velocities, but which are not part of the global parameter vector $\vec{P}$, and hence also not part of $\vec{Q}$.

All vector function terms may involve a delay function, i.e., an expression of the form delay(*x*,*τ*) with *τ*>0. It is therefore possible to address values of *x* computed in the earlier integration step at time *t*−*τ*, turning equation (3) into a delay differential equation (DDE). Note that *x* can be an arbitrarily complex expression.

In the general case of equation (3), not all species’ amounts can be computed by integrating the transformation $N\vec{\nu }$: the change of some model quantities may be given in the form of rate rules by function $\vec{g}(\vec{Q},t)$. Species whose amounts are determined by rate rules must not participate in any reaction and hence only have zero-valued corresponding entries in the stoichiometric matrix **N**. Thereby, the rate rule function $\vec{g}(\vec{Q},t)$ directly gives the rate of change of these quantities, and returns 0 for all others.

In addition, SBML introduces the concept of events ${\vec{f}}_{E}(\vec{Q},t)$ and assignment rules $\vec{r}(\vec{Q},t)$. An event can directly manipulate the value of several quantities, for instance, reduce the size of a compartment to a certain portion of its current size, as soon as a trigger condition becomes satisfied. An assignment rule also influences the absolute value of a subject quantity.

A further concept in SBML is that of algebraic rules, which are equations that must evaluate to zero at all times during the simulation of the model. These rules can be solved to determine the values of quantities whose values are not determined by any other construct. In this way, conservation relations or other complex interrelations can be expressed in a very convenient way. With the help of bipartite matching [29] and a subsequent conversion it is possible to turn algebraic rules into assignment rules and hence include these into the term $\vec{r}(\vec{Q},t)$. Such a transformation, however, requires symbolic computation and is thus a complicated endeavor.

When the system under study operates at multiple time scales, i.e., it contains a fast and a slow subsystem, a separation of the system is necessary, leading to differential algebraic equations (DAEs). Some species can be declared to operate at the system’s boundaries, assuming a constant pool of their amounts or concentrations. Care must also be taken with respect to the units of the species, because under certain conditions division or multiplication with the sizes of their surrounding compartments becomes necessary in order to ensure the consistent interpretation of the models. For all these reasons, solving equation (3) is much more complicated than computing the solution of the simple equation (2) alone.

From the perspective of software engineering, a strict separation of the interpretation of the model and the numerical treatment of the differential equation system is necessary to ensure that regular numerical methods can be used to solve equation (3). In order to efficiently compute this solution, multiple preprocessing steps are required, such as the conversion of algebraic rules into assignment rules, or avoiding repeated recomputation of intermediate results. The next sections will give a detailed explanation of the necessary steps to solve these systems and how to efficiently perform their numerical integration with standard numerical solvers.

### Initialization

**Figure 1:**
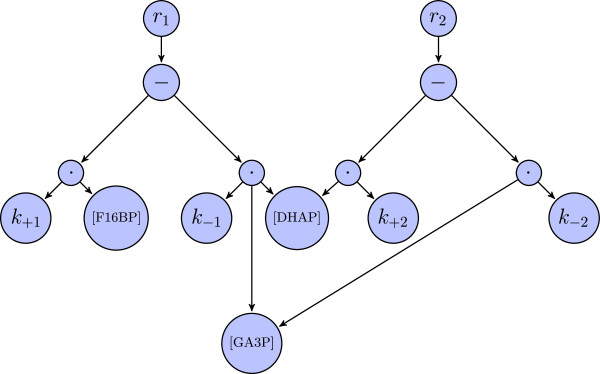
**Example for the creation of an abstract syntax graph of a small model.** This figure displays a unified representation of kinetic equations from an example model that consists of the following reactions: $R_{1}:\;\text{F1,6BP}⇌\text{DHAP}+\text{GA3P},\;R_{2}:\text{DHAP}⇌\text{GA3P}$. Both reactions are part of the glycolysis. The contained molecules are fructose 1,6-bisphosphate (F1,6BP), dihydroxyacetone phosphate (DHAP), and glyceraldehyde 3-phosphate (GA3P). Using the program SBMLsqueezer [31] the following mass action kinetics have been created: $\nu_{R_{1}}=k_{+1}\cdot [\;\text{F1,6BP}]-k_{-1}\cdot [\;\text{DHAP}]\cdot [\;\text{GA3P}],\;\nu_{R_{2}}=k_{+2}\cdot$ [ DHAP]−*k*_−2_·[ GA3P]. The nodes for [DHAP] and [GA3P] are only contained in the syntax graph once and connected to more than one multiplication node. This figure clearly indicates that the syntax graph is not a tree. As can be seen in this picture, the outdegree of syntax trees does not have to be binary.

At the beginning of the simulation the values of species, parameters and compartments are set to the initial values given in the model. All rate laws of the reactions, assignment rules, transformed algebraic rules (see below), initial assignments, event assignments, rate rules and function definitions are integrated into a single directed acyclic syntax graph. This graph is thus the result of merging the abstract syntax trees representing all those individual elements. Equivalent elements are only contained once. In comparison to maintaining multiple syntax trees, this solution significantly decreases the computation time needed for the evaluation of syntax graphs during the simulation. Figure 1 gives an example for such a syntax graph.

After the creation of this graph, the initial assignments and the assignment rules (including transformed algebraic rules) are processed and initial values defined by these constructs are computed.

### Solving algebraic rules

**Figure 2:**
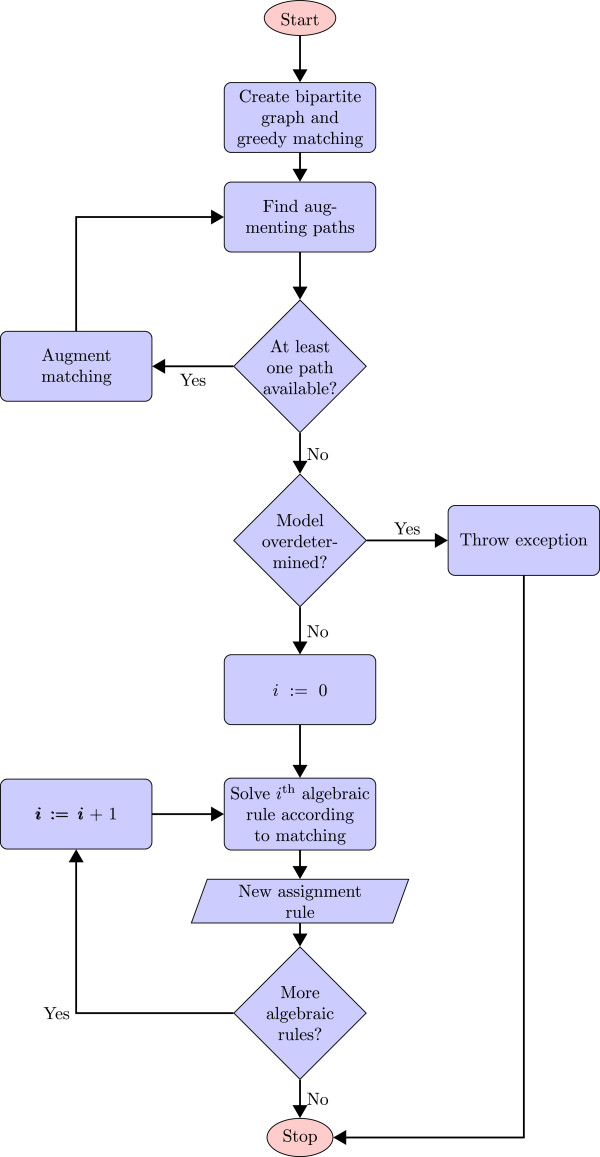
**Algorithm for transforming algebraic rules to assignment rules.** The first step is to decide whether the model is overdetermined by creating a matching between the equations and the variables of a model. For this purpose, an initial greedy matching is computed based on a bipartite constructed according to the SBML specifications. To obtain a maximal matching, augmenting paths are determined and the current matching is extended. If there are no augmenting paths available anymore, the computed matching is maximal. Having an unconnected equation vertex results in an overdetermined model. If the matching is not overdetermined, for each algebraic rule an assignment rule is generated. The left-hand side of each rule corresponds to the variable the respective algebraic rule has been matched to.

The most straightforward approach to deal with algebraic rules is to convert them to assignment rules, which can in turn be directly solved. In every equation of an algebraic rule, there should be at least one variable whose value is not yet defined through other equations in the model. This variable has to be determined for the purpose of interpreting the algebraic rule. At first, a bipartite graph is generated according to the SBML specifications [19-22]. This graph is used to compute a matching using the algorithm by Hopcroft and Karp [29]. The initial greedy matching is extended with the use of augmenting paths. This process is repeated until no more augmenting paths can be found. Per definition, this results in a maximal matching. As stated in the SBML specifications [19-22], if any equation vertex remains unconnected after augmenting the matching as far as possible, the model is considered overdetermined and thus is not a valid SBML model. If this is not the case, the mathematical expression of every algebraic rule is thereafter transformed into an equation with the target variable on its left-hand side, and hence fulfills the definition of an assignment rule. The left-hand side is represented by the respective variable vertex, to which the considered algebraic rule has been matched. Figure 2 displays the described algorithm in the form of a flow chart.

### Event handling

An event in SBML is a list of assignments that is executed depending on whether a trigger condition switches from *false* to *true*. In addition, SBML enables modellers to define a delay which may postpone the actual execution of the event’s assignments to a later point in time. With the release of SBML Level 3 Version 1, the processing of events has been raised to an even higher level of complexity: in earlier versions it was sufficient to determine, when an event triggers and when its assignments are to be executed. In Level 3 Version 1 only a few new language elements have been added, but these have a significant impact on how to handle events: for example, the order, in which events have been processed, used to be at programmer’s discretion in SBML Level 2, but in Level 3 Version 1 it is given by the event’s priority element. Coordinating the sequence, in which events are to be executed, has now become the crucial part of event handling. Furthermore, there exists the option to cancel an event during the time since its trigger has been activated and the actual time when the scheduler picks the event for execution. Events that can be cancelled after the activation of their triggers are called *nonpersistent*.

**Figure 3:**
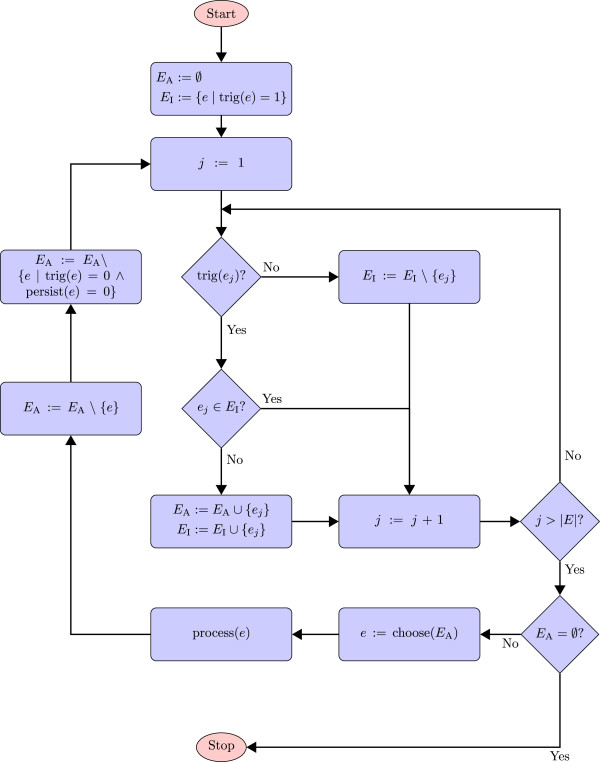
**Processing of events: simplified algorithm (handling of delayed events omitted).** At each iteration, the trigger conditions of active events *e*_a_∈*E*_A_ that are not persistent are checked. If the trigger condition of such an event has changed from *true* (1) to *false* (0), the event is removed from *E*_A_. The next step comprises the evaluation of the triggers of all events. If its trigger changes from *false* to *true*, an event is added to the set of active events *E*_A_. An event with its trigger changed from *true* to *false* is removed from the list of inactive events. After the processing of all triggers, the event *e* of highest priority in the set of active events is chosen for execution by the function choose(*E*_A_). Note that priorities are not always defined, or multiple events may have an identical priority. The function choose(*E*_A_) is therefore more complex than shown in this figure. The selected event is then processed, i.e., all of its assignments are evaluated, and afterwards the triggers of all events in *E* have to be evaluated again, because of possible mutual influences between the events. The algorithm proceeds until the set *E*_A_ of active events is empty.

At every time step, the events to be executed are a union of two subsets of the set of all events. On one hand, there are events whose triggers have been activated at the current time and which are to be evaluated without delay. On the other hand, there are events triggered at some time point before, and whose delay reaches till the current point in time. For every element of the resulting set of events, the priority rule must be evaluated. One event is randomly chosen for execution from all events of highest priority. In principle, all other events could be processed in the same manner, but the assignment of the first event can change the priority or even the trigger condition of the events that have not yet been executed. Therefore, the trigger of nonpersistent events and the priority of the remaining events have to be evaluated again. In this case, the event that has now the highest priority is chosen as next. This process must be repeated until no further events are left for execution. Figure 3 shows the slightly simplified algorithm for event processing at a specific point in time: Let *E* be the set of all events in a model, and *E*_I_ be the set of events whose trigger conditions have already been evaluated to *true* in previous time steps. We refer to elements within *E*_I_ as *inactive* events. We define the set *E*_A_ as the subset of *E* containing events whose trigger condition switches from *false* to *true* at the current time step *t*. At the beginning of the event handling, *E*_A_ is empty. We call an event *persistent*, if it can only be removed from *E*_A_ under the condition that all of its assignments have been evaluated. This means that a *nonpersistent* event can be removed from *E*_A_ when its trigger condition becomes *false* during the evaluation of other events. The function trig(*e*) returns 1 or 0 depending on whether or not the trigger condition of event *e*∈*E* is satisfied. Similarly, the function persist(*e*) returns 1 if event *e* is persistent, or 0 otherwise.

The interpretation of events is the most time consuming step of the integration procedure. This is why efficient and clearly organized data structures are required to ensure high performance of the algorithm.

### Time step adaptation considering events and the calculation of derivatives

**Figure 4:**
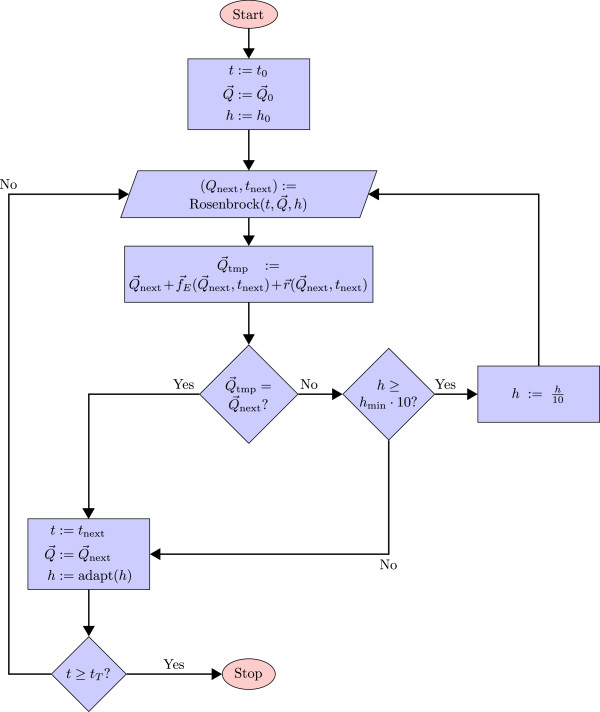
**Refined step-size adaptation for events.** For a certain time interval, the Rosenbrock solver (KiSAO term 33) always tries to increase time *t* by the current adaptive step size *h* and calculates a new vector of quantities ${\vec{Q}}_{\text{next}}$. After a successful step, the events and rules of the model are processed. If this causes a change in $\vec{Q}$, *h* is first decreased and the Rosenbrock solver then calculates another vector ${\vec{Q}}_{\text{next}}$ using this adapted step size. The precision of the event processing is therefore determined by the minimum step size *h*_min_. The adapt function is defined by Rosenbrock’s method [30].

The precise calculation of the time when events are triggered is crucial to ensure exact results of the numerical integration process. It could, for instance, happen that an event is triggered at time *t*_*τ*_, which is between the integration time points *t*_*τ*−1_ and *t*_*τ*+1_. When processing the events only at time points *t*_*τ*−1_ and *t*_*τ*+1_, it might happen that the trigger condition cannot be evaluated to *true* at neither of these time points. Hence, a numerical integration method with step-size adaptation is required in order to hit the correct time points. Rosenbrock’s method [30] can adapt its step size *h* if events occur (see Figure 4 for details). For a certain time interval [*t*_*τ*−1_,*t*_*τ*+1_] and the current vector $\vec{Q}$, Rosenbrock’s method determines the new value of vector $\vec{Q}$ at a point in time *t*_*τ*−1_+*h*, with *h*>0. If the error tolerance cannot be respected, *h* is reduced and the procedure is repeated.

After that, the events and the assignment rules are processed at the new point in time *t*_*τ*−1_+*h*. If the previous step causes a change in $\vec{Q}$, the adaptive step size is decreased by setting *h* to *h*/10 and the calculation is repeated until either the minimum step size is reached or the processing of events and assignment rules does not change $\vec{Q}$ anymore. Hence, the time at which an event takes place is precisely determined.

**Figure 5:**
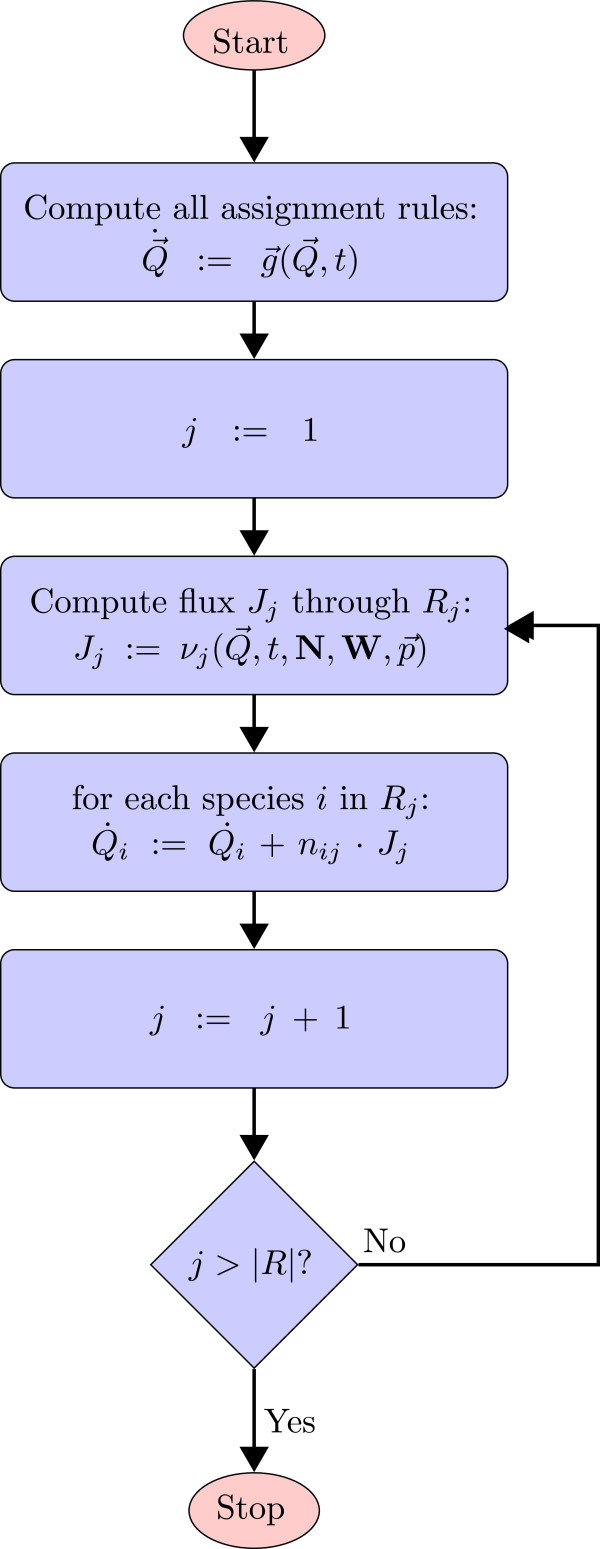
**Calculation of the derivatives at a specific point in time.** First, the vector for saving the derivatives of all quantities $\dot{\vec{Q}}$ is set to the null vector $\vec{0}$. Then the rate rules of the model are processed by solving the function $\vec{g}(\vec{Q},t)$, which can change $\dot{\vec{Q}}$ in some dimensions. After that for every reaction channel *R*_*j*_ its velocity *J*_*j*_ is computed. The derivatives of each species (with index *i*) participating in the currently processed reaction channel *R*_*j*_ are updated in each step adding the product of the stoichiometry *n*_*ij*_ and the reaction’s velocity *J*_*j*_. In this figure, the stoichiometric values *n*_*ij*_ in the matrix **N** are assumed to be constant for the sake of simplicity. These values can be variable. Before Level 3, SBML provided StoichiometryMath elements that could be used for a direct computation of the stoichiometry. In Level 3, the StoichiometryMath element has been removed and these values can be changed by treating them as the subject of assignment rules. In both cases, the values for *n*_*ij*_ have to be updated in each simulation step.

For given values $\vec{Q}$ at a point *t* in time the current vector of derivatives $\dot{\vec{Q}}$ is calculated as follows. First, the rate rules are processed $\dot{\vec{Q}}=\vec{g}(\vec{Q},t)$. Note that function $\vec{g}$ returns 0 in all dimensions in which no rate rule is defined. Second, the velocity *ν*_*i*_ of each reaction channel *R*_*i*_ is computed with the help of the unified syntax graph (e.g., Figure 1). The velocity functions depend on $\vec{Q}$ at time *t*. During the second step, the derivatives of all species that participate in the current reaction *R*_*i*_ need to be updated (see the flowchart in Figure 5).

### A reference implementation of the algorithm

**Figure 6:**
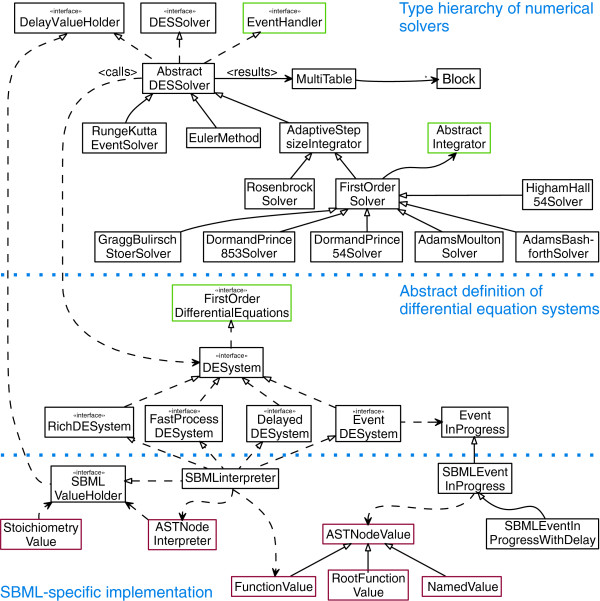
**Architecture of the Systems Biology Simulation Core Library (simplified).** Numerical methods are strictly separated from differential equation systems. The upper part displays the unified type hierarchy of all currently included numerical integration methods. The middle part shows the interfaces defining several special types of the differential equations to be solved by the numerical methods. The class SBMLinterpreter (bottom part) implements all of these interfaces with respect to the information content of a given SBML model. Similarly, an implementation of further data formats can be included into the library.

The algorithm described above has been implemented in Java™ and included into the Systems Biology Simulation Core Library. Figure 6 displays an overview of the software architecture of this community project, which has been designed with the aim to provide an extensible numerical backend for customized programs for research in computational systems biology. The SBML-solving algorithm is based on the data structures provided by the JSBML project [31]. With the help of wrapper classes several numerical solvers originating from the Apache Commons Math library [32] could be included into the project. In addition, the library provides an implementation of the explicit fourth order Runge-Kutta method, Rosenbrock’s method, and Euler’s method.

Due to the strict separation between numerical differential equation solvers, and the definition of the actual differential equation system, it is possible to implement support for other community standards, such as CellML [9].

In order to support the standard Minimum Information About a Simulation Experiment (MIASE) [33], the library also provides an interpreter of Simulation Experiment Description Markup Language (SED-ML) files [26]. These files allow users to store the details of a simulation, including the selection and all settings of the numerical method, hence facilitating the creation of reproducible results. A simulation experiment can also be directly started by passing a SED-ML file to the interpreter in this library. Each solver has a method to directly access its corresponding Kinetic Simulation Algorithm Ontology (KiSAO) term [34] to facilitate the execution of SED-ML files.

Many interfaces, abstract classes, and an exhaustive source code documentation in the form of JavaDoc facilitate the customization of the library. For testing purposes, the library contains a sample program that benchmarks its SBML interpreter against the entire SBML Test Suite version 2.3.2 [24].

### Benchmark and application to published models

The reference SBML implementation has successfully passed the SBML Test Suite [24] using the Rosenbrock solver. The results are shown in Table 1. All models together can be simulated within seconds, which means that the simulation of one SBML model takes only milliseconds on average, using regular desktop computers.

**Table 1:** Simulation of the models from the SBML Test Suite using the Rosenbrock solver

**Level**	**Version**	**Models**	**Correct simulations**	**Total running time (in s)**
1	2	252	252	2.9
2	1	885	885	6.8
2	2	1,041	1,041	6.8
2	3	1,041	1,041	6.8
2	4	1,043	1,043	6.8
3	1	1,077	1,077	38.5

This table shows the number of tested models and the total running times of the tests for all SBML levels and versions, where the time for reading the file has been excluded from the analysis. The measured elapsed time therfore gives the CPU time needed for the computation only (see Methods).

The total simulation time for all models in SBML Level 3 Version 1 is significantly higher than for the models in other SBML levels and versions. This can be explained by the fact that the test suite contains some models of this version whose evaluation requires a time-consuming processing of a large number of events. In particular, the simulation of model No. 966 of the SBML Test Suite, which is only provided in SBML Level 3 Version 1, takes 20s because it contains 23 events to be processed. Two events fire every 10^−2^ time units within the simulation time period of 1,000 time units. These events must therefore be evaluated thousandfold within the specified time interval. The evaluation of this model accounts for over 50% of the total simulation time for the models in SBML Level 3 Version 1.

An implementation of an SBML solver that passes the test suite should in principle also be capable of computing the solution of all models from BioModels Database, a resource that contains a collection of published and curated models. This online database currently provides neither reference data for the models, nor any settings for the numerical computation (such as step size, end time etc.). However, it offers pre-computed plots of the time courses for the vast majority of models. Therefore, while it cannot be directly used as a benchmark test, it can help checking that a solver implementation supports all features of many published models and that the algorithm always successfully terminates. The Systems Biology Simulation Core Library solves all curated models from BioModels Database (release 23, October 2012) without raising any errors, see Methods for details. These results suggest the reliability of the simulation algorithm described in this work.

In the following, we select two models that exhibit diverse features from this repository to illustrate the capabilities of this library: BioModels Database model No. 206 by Wolf *et al.*[35] and BioModels Database model No. 390 by Arnold and Nikoloski [36].

**Figure 7:**
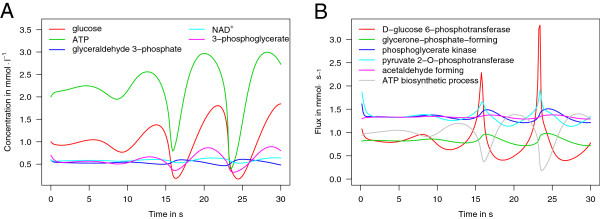
**Simulation of glycolytic oscillations.** This figure displays the results of a simulation computed with the Systems Biology Simulation Core Library based on model No. 206 from BioModels Database [35,37]. **A)** Shown are the changes of the concentration of the most characteristic intracellular metabolites 3-phosphoglycerate, ATP, glucose, glyceraldehyde 3-phosphate (GA3P), and NAD^+^ within yeast cells in the time interval [ 0, 30] seconds. **B)** This panel displays a selection of the dynamics of relevant fluxes (D-glucose 6-phosphotransferase, glycerone-phosphate- forming, phosphoglycerate kinase, pyruvate 2-O-phosphotransferase, acetaldehyde forming, ATP biosynthetic process) that were computed as intermediate results by the algorithm. The computation was performed using the Adams-Moulton solver [38] (KiSAO term 280) with 200 integration steps, 10^−10^ as absolute error tolerance and 10^−5^ as relative error tolerance. Due to the importance of feedback regulation the selection of an appropriate numerical solver is of crucial importance for this model. Methods without step-size adaptation, such as the fourth order Runge-Kutta algorithm (KiSAO term 64), might only be able to find a high-quality solution with an appropriate number of integration steps. The simulation results obtained by using the algorithm described in this work reproduces the results provided by BioModels Database.

The model by Wolf *et al.*[35] mimics glycolytic oscillations that have been observed in yeast cells. The model describes how the dynamics propagate through the cellular network comprising eleven reactions, which interrelate nine reactive species. Figure 7a displays the simulation results for the intracellular concentrations of 3-phosphogylcerate, ATP, glucose, glyceraldhyde 3-phosphate, and NAD ^+^: after an initial phase of approximately 15 s all metabolites begin a steady-going rhythmic oscillation. Changes in the dynamics of the fluxes through selected reaction channels within this model can be seen in Figure 7b.

**Figure 8:**
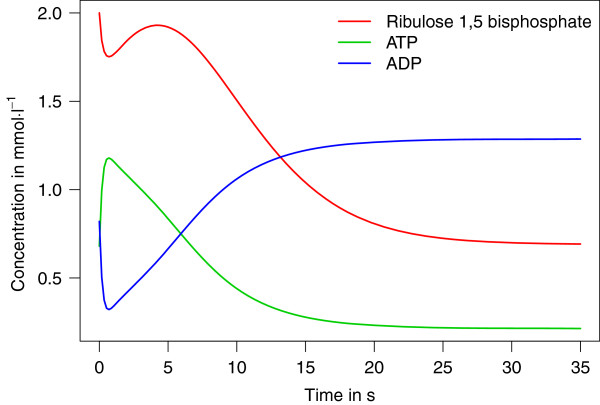
**Simulation of the Calvin-Benson cycle.** Another example of the capabilities of the Simulation Core Library has been obtained by solving model No. 390 from BioModels Database [36,37]. This figure shows the evolution of the concentrations of ribulose 1,5-bisphosphate, a key metabolite for CO_2_ fixation in the reaction catalyzed by ribulose-1,5-bisphosphate carboxylase oxygenase (RuBisCO), and the currency metabolites ATP and ADP during the first 35s of the photosynthesis. This model was simulated using Euler’s method (KiSAO term 30) with 200 integration steps.

By comparing a large collection of previous models of the Calvin-Benson cycle, Arnold and Nikoloski created a quantitative consensus model that comprises eleven species, six reactions, and one assignment rule [36]. All kinetic equations within this model call specialized function definitions. Figure 8 shows the simulation results for the species ribulose-1,5-bisphosphate, ATP, and ADP within this model. As in the previous test case, the dynamics computed by the Simulation Core Library reproduce the figures provided by BioModels Database.

### Comparison to existing solver implementations for SBML

In order to benchmark our software, we chose similar tools exhibiting the following features from the SBML software matrix [39]: 

• The last updated version was released after the final release of the specification for SBML Level 3 Version 1 Core, i.e., October 6^th^ 2010.

• Support for SBML Level 3.

• Open-source software

• No dependency on commercial products that are not freely available (e.g., MATLAB™ or Mathematica™)

The selected programs are in alphabetical order: BioUML [40], COPASI [41], iBioSim [42], JSim [43], LibSBMLSim [44], and VCell [45,46]. Table 2 summarizes the comparison of the most recent versions of all six programs. It should be mentioned that this comparison can only mirror a snapshot of the ongoing development process of all programs at the time of writing. An up-to-date comparison of the capabilities of SBML solvers can be found online [47].

**Table 2:** Comparison of SBML-capable simulators

**Program**	**Version**		**Difficult SBML**		**Fully SBML test**	**SED-ML**	**Programming**	**GUI**	**API**	**Platform**	**Comments**
			**elements**		**Suite compliant**		**language**		**access**		
		**Fast**	**Algebraic**	**Events**							
		**reactions**	**rules**								
BioUML	0.9.4	✓	✓	✓	✓	In *α*	Java	✓	JavaScript	Independent	
						version					
COPASI	4.9.45	–	–	(✓)	–	–	C++ (with	✓	✓	Windows, Mac OS X,	
							multiple bindings)			Linux, Solaris	
iBioSim	2.4.5	✓	✓	✓	✓	In *α*	Java, C	✓	(✓)	Windows, Mac OS X,	
						version				Linux (Fedora 17)	
JSim	2.10	–	✓	–	–	–	Java	✓	✓	Windows, Mac OS X,	
										Linux	
LibSBMLSim	1.1.0	✓	✓	✓	(✓)	–	C (with multiple)	–	✓	Windows, Mac OS X,	
							bindings)			Linux, Free BSD	
Simulation	1.3	✓	✓	✓	✓	✓	Java	–	✓	Independent	
core library											
VCell	5.0	✓	–	✓	–	–	Java frontend,	✓	–	Independent	Internet
							C/C++ server				connection
							backend				required

The table gives an overview about the most characteristic features of SBML-capable simulation programs (April 19^th^ 2013). It shows which programs support the SBML elements fast reactions, algebraic rules, and events. Another key point is whether all models of the most recent SBML Test Suite [24] can be correctly solved. Note that in the SBML Test Suite column, a dash means that *not all* of its models can be correctly solved, because not all SBML elements are supported. LibSBMLSim, which is a simulation API written in C, can only read models given in SBML Level 2 Version 4 and SBML Level 3 (indicated by the checkmark in brackets). Similarly, a dash in the column for events means that *not all* possible cases for this language element can be correctly solved. COPASI, for instance, supports events in SBML, but not all of the current constructs. It should be mentioned that not all programs primarily focus on the use of ODE solvers. In iBioSim, for instance, the stochastic analysis of SBML is more important [52]. Furthermore, some programs such as VCell or COPASI do not use SBML as their native format. BioUML, iBioSim, and the Systems Biology Simulation Core Library, are the only simulation tools from this selection that pass *all* models of the SBML Test Suite across all levels and versions of SBML. Most programs provide direct access to their API. COPASI, LibSBMLSim, and the Systems Biology Simulation Core Library have particularly been designed for the use as a solver backend. The program iBioSim can be executed in a script, e.g., for batch processing of multiple models.

### Limitations and perspective

The modifications done to the Rosenbrock solver enable a precise timing of events during simulation. However, this precise timing can lead to a noticeable increase in run-time when events are triggered in very small intervals, e.g., every 10^−3^ time units. This behavior can, for example, be observed in BioModels Database model No. 408 [48] (a model with three events). When the precise timing of events is not of utmost importance, a solver other than Rosenbrock can be chosen. Furthermore, there are plans to improve the runtime behavior of the Rosenbrock solver for the simulation of models containing events.

When dealing with stiff problems, Rosenbrock’s method is a good choice, because it is has been designed for stiff pODE. However, our experiments show, that the Rosenbrock solver can be inefficient for non-stiff problems in comparison to other solvers. This issue can lead to an increased run-time regarding large models such as model No. 235 of the BioModels Database, which contains 622 species that participate in 778 reactions, distributed accross three compartments [49]. In some cases, tuning the relative and absolute tolerance can help, but depending on the system’s structure, Rosenbrock’s method is sometimes stretched to its limits. The Runge-Kutta-Fehlberg method [50] (KiSAO term 86), which is included in iBioSim, shows also an increase in run-time concerning this model.

The performance of the Runge-Kutta-Fehlberg and Rosenbrock methods show, however, that simpler ODE solvers can have more difficulties with some biological models than more advanced solvers, such as CVODE from SUNDIALS[51] that can adapt to both non-stiff and stiff problems. The SUNDIALS library, which is incorporated into BioUML, can handle complicated pODE significantly better, but since it is not available under the LGPL and no open-source Java version of these solvers can currently be obtained, we disregarded its use.

Algebraic rules constitute an important problem for any implementation of the SBML standard. The unbound variable of each such equation can be efficiently identified [29], whereas the transformation of an algebraic rule into an assignment rule includes symbolic computation and is very difficult to implement. In some cases, such a transformation is not even possible. Alternatively, the current value of the free variable in an algebraic equation could, for instance, be identified using nested intervals. However, this approach consumes a significantly higher run-time, because the nested intervals would have to be re-computed at every time step, whereas the transformation approach considers every algebraic rule only once (during the initialization).

Since Level 3, SBML entails one further aspect: it is now possible to add additional features to the model by declaring specialized extension packages. The algorithm discussed in this paper describes the core functionality of SBML. The extension packages are very diverse, reaching the graphical representation [53], the description of qualitative networks, such as Petri nets [54], and many more. It is therefore necessary to separately derive and implement algorithms for the interpretation of individual SBML packages.

The agenda for the further development of the open-source project, the Systems Biology Simulation Core Library, includes the implementation of SBML extension packages, support for CellML, and the incorporation of additional numerical solvers. Contributions from the community are welcome.

## Conclusions

The aim of this work is to derive a formal description of the mathematics behind SBML together with an algorithm that efficiently solves it in terms of an ordinary differential equation framework. As an important design feature, the algorithm can be combined with existing numerical solvers in a plugin fashion. The Rosenbrock solver embodies a universal approach for simulation that can deal with stiff problems and precisely solve models containing arbitrary SBML elements. The description in this paper is intended to facilitate the implementation of the algorithm within specifically tailored programs.

Our tests indicate that at the moment only two other programs pass the entire test suite for all SBML levels and versions: BioUML, which is a workbench for modelling, simulation, and parameter fitting, and iBioSim. The reference implementation of the algorithm introduced in this work, the Systems Biology Simulation Core Library, is therefore the only API simulation library exhibiting this capability.

The Systems Biology Simulation Core Library is an efficient Java tool for the simulation of differential equation systems used in systems biology. It can be easily integrated into larger customized applications. For instance, CellDesigner [55] has already been using it since version 4.2 as one of its integral simulation libraries. The stand-alone application SBMLsimulator [56] provides a convenient graphical user interface for the simulation of SBML models and uses it as a computational back-end. The abstract class structure of the library supports the integration of further model formats, such as CellML, in addition to its SBML implementation. To this end, it is only necessary to implement a suitable interpreter class.

By including support for the emerging standard SED-ML, we hope to facilitate the exchange, archival and reproduction of simulation experiments performed using the Systems Biology Simulation Core Library.

## Methods

### Implementation

All the solver classes are derived from the abstract class AbstractDESSolver (Figure 6). Several solvers of the Apache Commons Math library (version 3.0) are integrated with the help of wrapper classes [32]. Numerical methods and the actual differential equation systems are strictly separated. The class MultiTable stores the results of a simulation within its Block data structures.

The abstract description of differential equation systems, with the help of several distinct interfaces, makes it possible to decouple them from a particular type of biological network. It is therefore possible to pass an instance of an interpreter for a respective model description format to any available solver. The interpretation of SBML models is split between evaluation of events and rules, computation of stoichiometric information, and computation of the current values for all model components (such as species and compartments).

For a given state of the ODE system, the class SBMLinterpreter, responsible for the evaluation of models encoded in SBML, returns the current set of time-derivatives of the variables. It is connected to an efficient MathML interpreter of the expressions contained in kinetic laws, rules and events (ASTNodeInterpreter). The nodes of the syntax graph for those expressions depend on the current state of the ODE system. If the state has changed, the values of the nodes have to be recalculated (see Results).

An important aspect in the interpretation of SBML models is the determination of the exact time at which an event occurs because this influences the precision of the system’s variables. To this end, we adjusted an implementation of the Rosenbrock solver [57], an integrator with an adaptive step size, to a very precise timing of the events. In addition to events, rules are also treated during integration. Basically, rules are treated like events that occur at every given point in time and are therefore processed in the same manner. For every object of the type AlgebraicRule, a new AssignmentRule object is generated by means of the preceding bipartite matching. They represent only temporary rules, that are incorporated in the simulation process but do not influence the model in the SBML file.

In the SBMLinterpreter, events are represented via an array containing one instance of EventInProgress for every event in the model. Thereby, the distinction between events with and without delays is made. Both types of events can be triggered multiple times before being executed. If no delay is defined, the assignments of the event are usually executed at the same point in time when the event has been triggered. However, when such an event is cancelled by other events, all of its assignments are also cancelled before execution. An event with delay can produce multiple further assignments within the time frame between the trigger time and the actual execution time. In order to deal with delayed events, the class SBMLEventInProgressWithDelay keeps track of this via a list containing the points in time, at which the respective event has to be executed. When events are triggered more than once before execution, they have to be sorted in ascending order by their delay. This is neccesary, because in this case the delay of the very same event may vary.

When the SBMLinterpreter is processing events with priority, the events with the highest priority are stored in a list until one of them is selected for execution. Technically, the method of choice for the organization of such priority queues would apply a binary max heap data structure instead. The root of the heap represents the largest value in the heap. After its extraction, the heap property is restored so that the next largest value is moved to the root. However, as stated in Results, the execution of one event can influence the priority of the remaining events. It can possibly happen that many priorities simultaneously change, whereby the standard method to restore the max heap characteristic after extraction is not sufficient anymore. For this reason, we disregarded the use of more complex data structures for the current implementation.

Since SBML Level 2 Version 1, it has also become possible to create user-defined functions. These function definition objects contain lambda calculus including an optional list of arguments together with the actual mathematical expression of the function. During the initialization phase, function definitions are also incorporated into the abstract syntax graph (Figure 1). For each function definition, its arguments defined in its lambda expression are mapped to their corresponding nodes in the abstract syntax graph. The evaluation of a syntax graph node with a user-defined function consists of several steps. The arguments are evaluated and then passed to their corresponding node in the graph via the mapping established before. After this step the nodes representing arguments have a specific value attached to them. Finally, the complete abstract syntax graph can be evaluated. Care must be taken, because several function definitions may have arguments with identical identifiers. All possible naming conflicts must be preempted.

As part of the calculation of reaction velocities, the StoichiometryMath construct allows a dynamic change of a reaction’s stoichiometry over the course of the simulation. Since SBML Level 3 Version 1, the stoichiometry of a reaction can be directly altered, because it is now possible to address the identifier of a SpeciesReference as the target variable within rules or events. The SBMLinterpreter class flags reactions with changing stoichiometry during initialization and evaluates the corresponding abstract syntax graph anew if the stoichiometry is needed for calculation.

The constraints introduce assumptions about a model’s behavior. Similar to the trigger of events, the abstract syntax graph of each constraint is evaluated at every time step. In case of a violation the SBMLinterpreter generates an instance of ConstraintEvent that is then processed by the corresponding ConstraintListener class. The user is informed about the constraint upon its violation via the standard Java Logger. The output message includes the point in simulation time and the message of the constraint. In addition, more advanced user-defined implementations of ConstraintListener can be added to the SBMLinterpreter, for instance, to notify a GUI about violations or display the associate messages in a more user-friendly way.

SED-ML support is enabled by inclusion of the jlibsedml library [58] in the binary download. Clients of the Systems Biology Simulation Core Library can choose to use the jlibsedml API directly, or access SED-ML support via facade classes in the org.simulator.sedml package that do not require direct dependencies on jlibsedml in their code.

### Default settings and configuration

The standard preferences for simulating an SBML model consist of the Rosenbrock solver with an absolute tolerance of 10^−12^ and a relative tolerance of 10^−6^. On the basis of our experiments, this setup can handle most of the problems without further tuning. The Rosenbrock solver with its adaptive step size is the most effective solver in this library for stiff pODE. Nevertheless, the user has the possibility the choose another solver for integration. According to the SBML specifications, a model has to be simulated starting at time point 0.0. Since this library is not limited to SBML, the solvers also accept arbitrary start times. The user has also the possibility to specify the end of the simulation. Modifying the relative and absolute tolerance can increase the accuracy of the results or decrease computation time.

### Simulation of models from BioModels Database

All 424 curated models from BioModels Database [11] (release 23, October 2012) have been simulated with identical settings, as suggested by Bergmann *et al.*[25]: time interval [0,10], the Rosenbrock solver, 10^−6^ as relative and 10^−12^ as absolute tolerance, and a step size of 0.01 time units. For the models No. 234 [59] and No. 339 [60] from BioModels Database the absolute tolerance had to be set to 10^−10^ in order to achieve the necessary accuracy and to avoid that the algorithm surpasses its minimal step size. On a sample basis, individual models have been selected and manually compared to the pre-computed plots provided by BioModels Database in order to check the correctness of the simulation results.

### Simulation of the SBML test suite

The models from SBML Test Suite version 2.3.2 [24] were first simulated with the Rosenbrock solver, 10^−6^ as relative and 10^−12^ as absolute tolerance. For six models (No. 863, 882, 893, 994, 1109, and 1121) we had to set the relative tolerance to 10^−8^ in order to simulate as accurately as desired. For three other models (No. 872, 987, 1052) the relative tolerance even had to be set to 10^−12^ and the absolute tolerance to 10^−14^.

### Hard- and software configuration

For all run-time tests, an Intel ^*Ⓡ*^ Core™ i5 CPU with 3.33GHz and 4GB RAM was used with Microsoft ^*Ⓡ*^ Windows ^*Ⓡ*^ 7 (Version 6.1.7600) as operating system and Java Virtual Machine version 1.6.0_25.

The Systems Biology Simulation Core Library was also successfully tested under Linux (Ubuntu version 10.4) and Mac OS X (versions 10.6.8 and 10.8.2).

## Availability and requirements

The current version of Systems Biology Simulation Core Library is available at the project’s homepage. The entire project, including source code and documentation, several versions of jar files containing only binaries, binaries together with source code, can be downloaded, optionally also as a version including all required third-party libraries.

**Project name:** Systems Biology Simulation Core Library

**Project homepage:**http://simulation-core.sourceforge.net

**Operating systems:** Platform independent, i.e., for all systems for which a JVM is available.

**Programming language:** Java™

**Other requirements:** Java Runtime Environment (JRE) 1.6 or above

**License:** GNU Lesser General Public License (LGPL) version 3

## Endnote

^a^ More than 250 available programs now support the SBML data format (April 19^th^ 2013).

## Abbreviations

ADP: Adenosine diphosphate; API: Application programing interface; ATP: Adenosine-5’-triphosphate; DHAP: Dihydroxyacetone phosphate; DAE: Differential algebraic equation; DDE: Delay differential equation; F1,6BP: Fructose 1,6-bisphosphate; GA3P: Glyceraldehyde 3-phosphate; GUI: Graphical user interface; JAR: Java archive file; JDK: Java development kit; JRE: Java runtime environment; JVM: Java virtual machine; KiSAO: Kinetic simulation algorithm Ontology; MIASE: Minimum information about a simulation experiment; LGPL: GNU lesser general public license; ODE: Ordinary differential equation; RuBisCO: Ribulose-1,5-bisphosphate carboxylase oxygenase; NAD+: Nicotinamide adenine dinucleotide; SBML: Systems biology markup language.

## Competing interests

The authors declare that they have no competing interests.

## Authors’ contributions

RK and AlD contributed equally, implemented the majority of the source code, and declare shared first authorship. MJZ and HP designed and implemented the abstraction scheme between solvers and ODE systems. NR and NLN designed, implemented, and coordinated the data structures for a smooth integration of JSBML. RA implemented support for SED-ML. AT and AF incorporated the Simulation Core Library into CellDesigner. NH created mathematical models which include several SBML features to test the integration with CellDesigner. AnD initialized and coordinated the project, drafted the manuscript, and supervised the work together with AZ. All authors contributed to the implementation, read and approved the final manuscript.
